# Predicting pulmonary hypertension with standard computed tomography pulmonary angiography

**DOI:** 10.1007/s10554-015-0618-x

**Published:** 2015-02-17

**Authors:** Onno A. Spruijt, Harm-Jan Bogaard, Martijn W. Heijmans, Rutger J. Lely, Mariëlle C. van de Veerdonk, Frances S. de Man, Nico Westerhof, Anton Vonk-Noordegraaf

**Affiliations:** 1 Department of Pulmonology, VU University Medical Center, de Boelelaan 1117, ZH 4F-010, 1081 HV Amsterdam, The Netherlands; 2 Department of Epidemiology and Biostatistics, VU University Medical Center, Amsterdam, The Netherlands; 3 Department of Radiology, VU University Medical Center, Amsterdam, The Netherlands; 4 Department of Physiology, VU University Medical Center, Amsterdam, The Netherlands

**Keywords:** Right ventricle, Precapillary PH, CT, Decision curve analysis

## Abstract

The most common feature of pulmonary hypertension (PH) on computed tomography pulmonary angiography (CTPA) is an increased diameter-ratio of the pulmonary artery to the ascending aorta (PA/AA_AX_). The aim of this study was to investigate whether combining PA/AA_AX_ measurements with ventricular measurements improves the predictive value of CTPA for precapillary PH. Three predicting models were analysed using baseline CTPA scans of 51 treatment naïve precapillary PH patients and 25 non-PH controls: model 1: PA/AA_AX_ only; model 2: PA/AA_AX_ combined with the ratio of the right ventricular and left ventricular diameter measured on the axial view (RV/LV_AX_); model 3: PA/AA_AX_ combined with the RV/LV-ratio measured on a four chamber view (RV/LV_4CH_). Prediction models were compared using multivariable binary logistic regression, ROC analyses and decision curve analyses (DCA). Multivariable binary logistic regression showed an improvement of the predictive value of model 2 (−2LL = 26.48) and 3 (−2LL = 21.03) compared to model 1 (−2LL = 21.03). ROC analyses showed significantly higher AUCs of model 2 and 3 compared to model 1 (*p* = 0.011 and *p* = 0.007, respectively). DCA showed an increased clinical benefit of model 2 and 3 compared to model 1. The predictive value of model 2 and 3 were almost equal. We found an optimal cut-off value for the RV/LV-ratio for predicting precapillary PH of RV/LV ≥ 1.20. The predictive value of CTPA for precapillary PH improves when ventricular and pulmonary artery measurements are combined. A PA/AA_AX_ ≥ 1 or a RV/LV_AX_ ≥ 1.20 needs further diagnostic evaluation to rule out or confirm the diagnosis.

## Introduction

Pulmonary hypertension (PH) is defined as an increase in mean pulmonary artery pressure (mPAP) above 25 mmHg [1]. Irrespective of the exact cause, the condition leads to right heart failure and finally death [2].

Most PH patients are diagnosed by the time their disease is in an advanced stage [3, 4]. The non-specific nature of symptoms at presentation (exercise-induced dyspnea, fatigue) leads to failure of physicians to recognize the disease and an undesirable late diagnosis. [4–7]. Early detection of PH and a timely initiation of treatment can significantly improve the clinical outcome [8–10]. A unique opportunity for an earlier diagnosis of PH is provided when a standard non-ECG gated computed tomography pulmonary angiography (CTPA) is performed to evaluate a patient presenting with shortness of breath. To the attentive radiologist, CTPA may provide important clues towards a diagnosis of PH.

An intensively studied feature to predict PH on CTPA is an increased diameter ratio of the pulmonary artery (PA) to ascending aorta (AA) [11–17]. Studies showed that this parameter has a sensitivity of 58–87 % for the diagnosis of PH. A way to improve the diagnostic sensitivity is to add information on the structure of the heart.

The clinical value of the ratio of the transverse diameter of the right ventricle (RV) and the left ventricle (LV) measured on the axial (AX) view and on a manually reconstructed four chamber (4CH) view is known as a typical sign of RV failure in acute pulmonary embolism [18, 19]. One study measured the RV/LV diameter ratio on the axial view in mainly post-capillary PH patients and found a sensitivity of 86 % [16]. It is unknown whether adding ventricular measurements to the PA/AA-ratio improves the diagnostic model of CTPA for precapillary PH.

Therefore, the aim of our study is to investigate whether combining PA measurements with ventricular measurements improves the predictive value of CTPA for precapillary PH.

## Methods

### Study subjects

The PH center in the VU University Medical Center is a tertiary referral center for PH patients in the Netherlands. From a large database of subjects who had been referred to the VU University Medical Center from 2002 through 2012 for the evaluation of pulmonary hypertension, we retrospectively, randomly selected treatment naïve precapillary PH patients. Only subjects in whom both a baseline right heart catheterization and baseline CTPA were performed, were included in this study. In total, 51 precapillary PH patients were randomly selected. Precapillary PH was diagnosed according to the World Health Organization guidelines (mPAP >25 mmHg and a pulmonary arterial wedge pressure ≤15 mmHg) [1].

25 subjects who were referred to our center for suspected PH and who appeared to have normal PA pressures during right heart catheterization and without a history of left heart disease, were randomly chosen and used as controls.

The study was approved by The Medical Ethics Review Committee of the VU University Medical Center. The study does not fall within the scope of the Medical Research Involving Human Subjects Act (WMO). Therefore, the study was approved without requirement of a consent statement.

### CTPA image acquisition

CTPA studies of the entire chest were performed on either a 4-slice multi-detector CT system (Somatom Volume Zoom, Siemens, Erlangen, Germany) or a 64-slice multi-detector CT system (Somatom Sensation, Siemens, Erlangen, Germany). 18 CTPA studies were performed on the 4-slice CT system and 58 CTPA studie were performed on the 64-slice CT system. The Dose Length Product (DLP) was 266 ± 118 mGy cm.

For the 4-slice multi-detector CT scanning parameters were 140 kV and 100 m as with dose modulation at a slice collimation of 4 × 1.0 mm, a rotation time of 0.5 s and a pitch of 1.25 out of which 1.5 mm axial slices with 1 mm reconstruction increment were reconstructed. The series were acquired using bolus tracking within the PA at maximum inspiration after intravenous injection (4 ml/s) of 100 ml of a low-osmolar, non-ionic contrast agent with iodine concentration of 300 mg/ml (Ultravist-300 Iopromide; Bayer Pharma AG, Berlin, Germany), using an injection pump through an 18 g cannula preferably in the right antecubital vein.

For the 64-slice multidetector CT, a slice collimation of 32 × 0.6 mm, a rotation time of 0.33 s and a pitch of 0.75 was used. The series were acquired using a test bolus (30 ml at 6 ml/s) with tracking in the PA and a scan bolus with calculated delay at maximum inspiration after intravenous injection (≤60 ml at 6 ml/s) of a low-osmolar, non-ionic contrast agent with a iodine concentration of 300 mg/ml (Ultravist-300 Iopromide; Bayer Pharma AG, Berlin, Germany), using an injection pump through an 18 g cannula mostly in the right antecubital vein.

### CTPA image analyses

CTPA studies were analyzed using a Sectra PACS IDS7 workstation. Measurements were performed by an investigator from the department of pulmonary diseases under supervision of a radiologist with special interest in thorax imaging. Intraobserver variability was tested by repeated measurements in 10 CT studies. To test interobserver variability, measurements were repeated in 20 CT studies by another investigator from the same department. Both observers were blinded to patients’ medical history, hemodynamic data and diagnosis.

### CTPA parameters

**Fig. 1:**
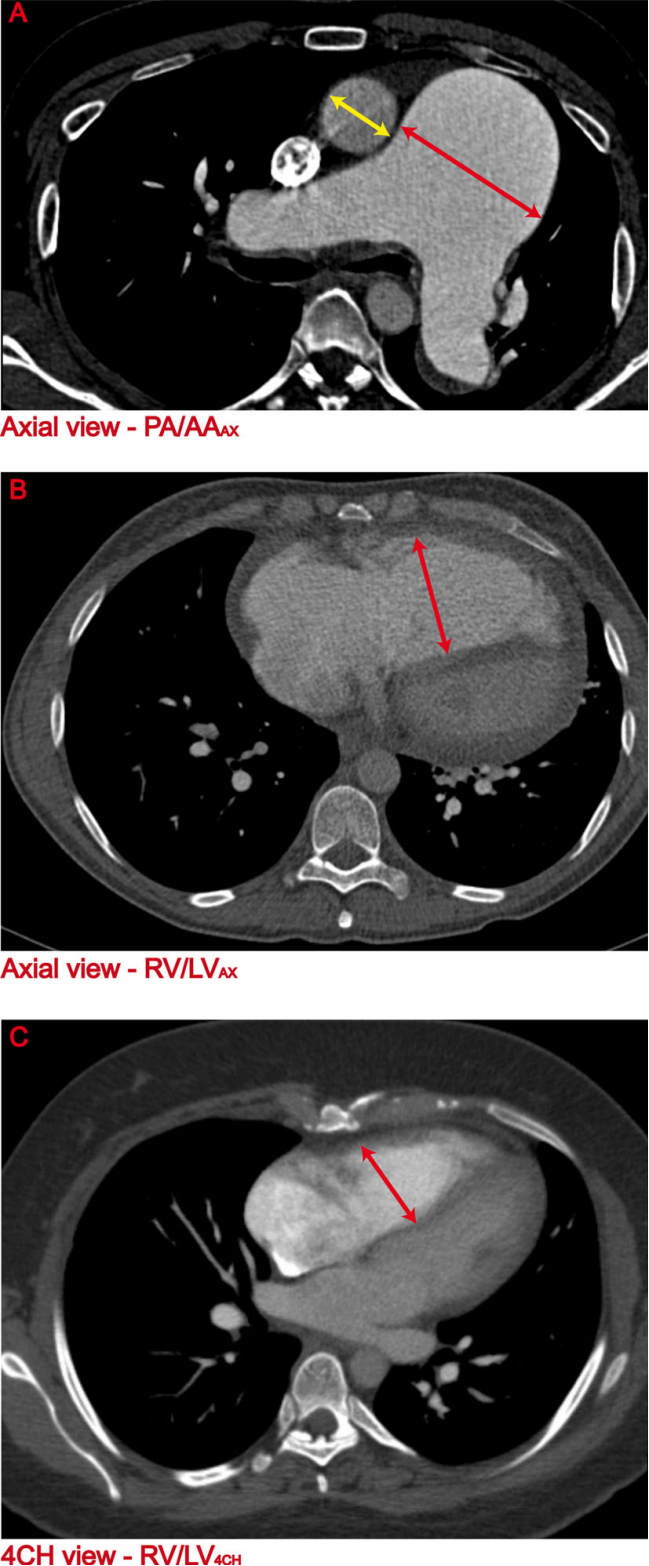
CTPA parameters **a** Pulmonary artery (PA) and ascending aorta (AA) ratio (PA/AA_AX_) on an axial view at the level of the bifurcation of the pulmonary trunk. **b** Right ventricle (RV) and left ventricle (LV) ratio (RV/LV_AX_) on an axial view. The RV diameter is measured perpendicular to the long axis of the heart. The LV diameter is not measured in this image, since the maximum diameter of the LV is not necessarily on the same image **c** RV/LV_4CH_ on a four chamber (4CH) view

PA/AA_AX_—Maximum diameters of the main PA and AA were obtained at the level of the bifurcation of the pulmonary trunk according to previous studies [11, 12]. PA and AA measurements were done on the same image in the axial view (Fig. 1a). Afterwards the PA/AA ratio was calculated.

RV/LV_AX_—Maximum transverse diameters of the RV and LV, defined as the widest distance of the endocardium between the interventricular septum and the free wall, were measured in the axial plane perpendicular to the long axis of the heart. Maximum diameters of the RV and LV were not necessarily obtained from the same image. Subsequently the RV/LV ratio was calculated (Fig. 1b).

RV/LV_4CH_—Multitplanar reconstruction (MPR) was used to manually reconstruct a 4CH view in the same manner as described earlier [18, 20]. Similar to the ventricular measurements in the axial view, the maximum transverse diameters of the RV and LV were obtained from the 4CH view and the RV/LV ratio was calculated. Again maximum diameters of the RV and LV were not necessarily acquired from the same image (Fig. 1c).

### Statistical analysis

Continuous data are presented as mean ± standard deviation (SD) and absolute numbers for categorical variables. Differences between mean values from precapillary PH patients and control subjects were analyzed using the unpaired Student t test (variables with a normal distribution) or Mann–Whitney U tests (variables not normally distributed). Intra and- interobserver variability of the three CTPA parameters were analyzed using simple linear regression analysis.

Univariable binary logistic regression analysis was used to test the predictive value of the three different CTPA parameters separately for precapillary pulmonary hypertension.

To test whether adding ventricular measurements to the PA/AA_AX_-ratio would improve the diagnostic model of CTPA for precapillary pulmonary hypertension, we compared three different diagnostic models: Model 1: PA/AA_AX_ (standard); Model 2: PA/AA_AX_ + RV/LV_AX_; and Model 3: PA/AA_AX_ + RV/LV_4CH_ (Table 1).

**Table 1:** Prediction models

Prediction models	
Model 1	PA/AA_AX_
Model 2	PA/AA_AX_ + RV/LV_AX_
Model 3	PA/AA_AX_ + RV/LV_4CH_

*PA/AA*_*AX*_ ratio between PA and AA, *RV/LV*_*AX*_ ratio between RV and LV in the axial plane *RV/LV*_*4CH*_ ratio between RV and LV in the 4CH view

The statistical approach to test the predictive value for precapillary PH of the three diagnostic models contained three different steps.

First we tested the predictive value of the three different models using multivariable binary logistic regression analysis. Second, the predictive value of the three different diagnostic models were tested using the area under the curve (AUC) derived from the Receiver Operating Characteristic curves. The AUC from the different models were compared using the DeLong method.

Third, to test the predictive value of the different diagnostic models within the clinical context of this study, we used decision curve analysis (DCA). With decision curve analysis it is possible to evaluate the clinical net benefit of the different prediction models [21, 22]. The net benefit is defined as the sum of benefits (true positives) minus the harms (false positives). Importantly, the threshold probability of the outcome determines the weights given to the true positives and false positives. The threshold probability is defined as the minimum probability of precapillary PH where a physician would decide to act. In this study it means that on the basis of the CTPA scan, it is decided to do further diagnostic tests to confirm the diagnosis. Since the exact threshold probability is unknown and will vary among physicians, we calculated the net benefit over a variety of probabilities. These net benefits can be calculated from the net benefit when nobody has precapillary PH (no positives) or from the net benefit when everybody has precapillary PH (no negatives). In this study, we focused on a range of low threshold probabilities (1–20 %) since the weight assigned to false negatives (missing the diagnosis) is considerably larger than to false positives (further diagnostic evaluation).

For clinical purposes of the diagnostic models, a cut-off value to define precapillary PH is demanded. An established cut-off value to define PH is PA/AA_AX_ > 1 [12]. A well-recognized cut-off value for the RV/LV-ratio is lacking. A frequently applied method for determining a cut-off value is calculation of the Youden Index, which is the cut-off value belonging to the highest sum of the combination of sensitivity and specificity, derived from the ROC-analysis. Since this cut-off value is not necessarily the optimal cut-off value within the clinical context, we chose a range of cut-off values to determine an optimal cut-off value.

Statistical analyses were performed using SPSS (version 20.0, SPSS, inc, Chicago, Illinois) and R (R Foundation for Statistical Computing, Vienna, Austria, 2013). *P* values < 0.05 were considered statistically significant.

## Results

### Baseline characteristics

Baseline characteristics of both groups are summarized in Table 2. Between groups there were expected differences in mPAP, pulmonary vascular resistance (PVR), right atrial pressure (RAP) and cardiac output (CO). The average interval time between the baseline right heart catherization and CTPA in the precapillary PH group was 16 ± 7  and 15 ± 5 days in the control group. Mean values of all three CTPA parameters were significantly different between precapillary PH patients and controls (Table 3).

**Table 2:** Baseline characteristics

	PH (N = 51)	Controls (N = 25)
Gender	71 % female	76 % female
Age (years)	56 ± 16	55 ± 15
Precapillary PH		
PAH	41	
CTEPH	10	
mPAP (mmHg)	48 ± 16	16 ± 4*
PAWP (mmHg)	7 ± 3	6 ± 3
PVR (Dyne.s/cm^5^)	774 ± 452	126 ± 70*
RAP (mmHg)	8 ± 5	3 ± 2*
CO (L/min)	5.1 ± 0.3	6.9 ± 0.4*

*IPAH* idiopathic pulmonary arterial hypertension, *CTEPH* chronic trombo-embolic pulmonary hypertension, *mPAP* mean pulmonary artery pressure, *PAWP* pulmonary artery wedge pressure, *PVR* pulmonary vascular resistance, *RAP* right atrial pressure, *CO* cardiac output

* *p* < 0.05 compared with the PH group

**Table 3:** CTPA parameters

CTPA parameters	PH	Controls
PA/AA_AX_	1.20 ± 0.30	0.85 ± 0.13*
RV/LV_AX_	1.62 ± 0.42	1.00 ± 0.20*
RV/LV_4CH_	1.65 ± 0.42	1.00 ± 0.18*

Mean values ± SD

*PA/AA*_*AX*_ ratio between PA and AA *RV/LV*_*AX*_ ratio between RV and LV in the axial plane *RV/LV*_*4CH*_ ratio between RV and LV in the 4CH view

* *p* < 0.05 compared with the PH group

### Intra- and interobserver vatiability

Intra- and interobserver variability was tested with simple linear regression and showed good agreement for all three parameters (*Intra:* PA/AA_AX_: β = 0.974 *p* < 0.001; RV/LV_AX_: β = 0.958 *p* < 0.001; RV/LV_4CH_: β = 0.896 *p* = 0.001. *Inter:* PA/AA_AX_: β = 0.971 *p* < 0.001; RV/LV_AX_: β = 0.965 *p* < 0.001; RV/LV_4CH_: β = 0.930 *p* < 0.001).

### Univariable and multivariable binary logistic regression analysis

Univariable binary logistic regression analysis showed that all three CTPA parameters were predictors of precapillary PH (Table 4). Multivariable binary logistic regression analysis showed an improvement of the predictive value for precapillary PH of model 2 (−2LL = 26.48) and 3 (−2LL = 21.03) compared with model 1 (−2LL = 56.56) and showed a slightly better predictive value of model 3(−2LL = 21.03) compared to model 2(–2LL = 26.48) (Table 5). A multivariate model with all three CTPA parameters was not possible because the correlation between RV/LV_AX_ and RV/LV_4CH_ was too strong (multicollinearity, VIF = 6.5).

**Table 4:** Univariable binary logistic regression analysis

CTPA parameters	−2LL	B	OR	95 % C.I.	*p* value
PA/AA_AX_	56.56	1.19	3.27	1.78–6.03	*p* < 0.001
RV/LV_AX_	47.22	0.82	2.26	1.51–3.39	*p* < 0.001
RV/LV_4CH_	44.77	0.86	2.37	1.51–3.71	*p* < 0.001

*B* beta, *OR* odds ratio, *95* *% C.I*. 95 % confidence interval

**Table 5:** Multivariate binary logistic regression analysis

Prediction models		−2LL	B	OR	95 % C.I.	*p* value
Model 1	PA/AA_AX_	56.56	1.19	3.27	1.78–6.03	*p* < 0.001
Model 2	PA/AA_AX_	26.48	1.79	5.99	1.67–21.45	*p* = 0.006
	RV/LV_AX_		0.82	2.28	1.37–3.78	*p* = 0.001
Model 3	PA/AA_AX_	21.03	2.40	10.98	1.73–69.52	*p* = 0.011
	RV/LV_4CH_		1.12	3.07	1.46–6.46	*p* = 0.003

*−2LL* = log-likelihood statistic, *B* beta, *OR* odds ratio, *95* *% C.I.* 95 % confidence interval

### ROC analysis

**Fig. 2:**
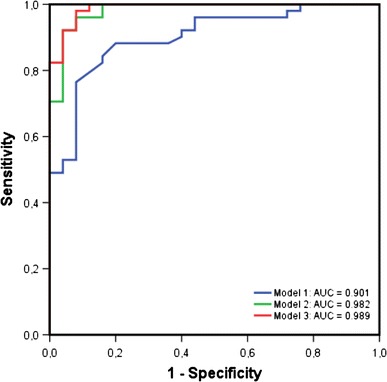
Area Under the Curve (AUC) of the three different models. *Blue line* = model 1, *Green line* = model 2, *Red line* = model 3

The AUC of the three different models are shown in Fig. 2. The AUC of model 2 and 3 were significantly higher than the AUC of model 1 (*p* = 0.011 and *p* = 0.007, respectively). There was no significant difference in the AUC between model 2 and 3 (*p* = 0.266).

### Decision curve analysis

**Fig. 3:**
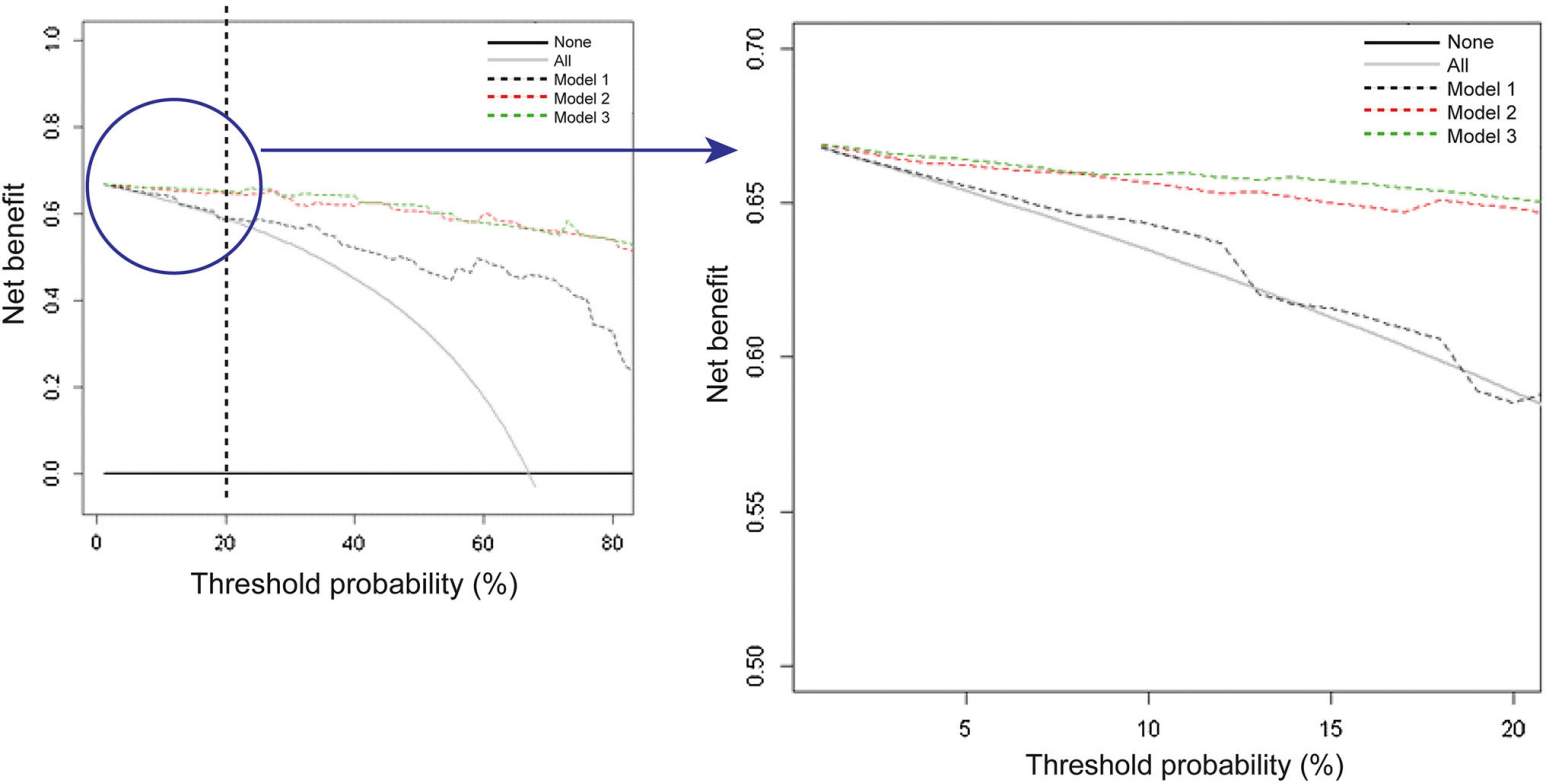
Decision curve analysis. Decision curve analysis of the three models to predict the presence of precapillary PH. On the right an expended view of the curves at low threshold probabilities, ranging from 0 to 20 %

The DCA curves of the three models are illustrated in Fig. 3. The black line represents the net benefit at different threshold probabilities if we would not use any model and decide that nobody has precapillary PH (no positives). Since the net benefit is defined as the sum of the true positives minus the false positives, the net benefit is zero at the entire range of threshold probabilities. The grey line represent the net benefit if we decide that everybody has precapillary PH (no negatives) and any of the models would not be used. We determined, at a range of low threshold probabilities (0–20 %), the net benefit of the three diagnostic models with respect to calling everybody a precapillary PH patient (grey line).

Results are summarized in Table 6. The net benefit of model 2 and 3 was, over the entire range of low threshold probabilities, better than the net benefit of model 1, with a decrease of up to 25 false positive patients without an increase in false negative patients. The net benefit of model 3 was also slightly better than model 2.

**Table 6:** Net benefits(NB) of model 1, 2 and 3

Threshold probability %	False Positives	NB PH all	NB Model 1: PA/AA_AX_	Delta NB	Decrease in false positives (per 100 patients) without an increase in false negatives
1	25	0.6677299	0.6677299	0.0000000	0
2	24	0.6643394	0.6646079	0.0002685	1
5	22	0.6537396	0.6558172	0.0020776	4
10	19	0.6345029	0.6432749	0.0087720	8
15	18	0.6130031	0.6160991	0.0030960	2
20	18	0.5888158	0.5855263	−0.0032895	−1
Threshold probability %	False Positives	NB PH all	NB Model 2: PA/AA_AX_ + RV/LV_AX_	Delta NB	Decrease in false positives (per 100 patients) without an increase in false negatives
1	18	0.6677299	0.6686603	0.0009304	9
2	17	0.6643394	0.6664877	0.0021482	11
5	13	0.6537396	0.6620499	0.0083102	16
10	10	0.6345029	0.6564328	0.0219298	20
15	9	0.6130031	0.6501548	0.0371517	21
20	7	0.5888158	0.6480263	0.0592105	24
Threshold probability %	False Positives	NB PH all	NB Model 3: PA/AA_AX_ + RV/LV_4CH_	Delta NB	Decrease in false positives (per 100 patients) without an increase in false negatives
1	16	0.6677299	0.6689261	0.0011962	12
2	14	0.6643394	0.6672932	0.0029538	14
5	10	0.6537396	0.6641274	0.0103878	20
10	8	0.6345029	0.6593567	0.0248538	22
15	6	0.6130031	0.6571207	0.0441176	25
20	6	0.5888158	0.6513158	0.0625000	25

The net benefit (NB) is calculated as: NB = (true positives/n)−[(false positives/n) × (Pt/(1-Pt)]. Subsequently, the decrease in false positives per 100 patients without an increase in false negatives is calculated as: (NB_model_−NB_all_) × 100(Pt/1-Pt)

*PT* threshold probability [21, 22]

### Cut-off value

To find an optimal cut-off value for defining precapillary PH, we analyzed a range of cut-off values which are summarized in Table 7. Since the weight assigned to false-negatives is larger than to false-positives, we looked for a cut-off value with a high sensitivity and negative predictive value, in combination with a relatively high specificity. Therefore, we chose as an optimal cut-off value for the RV/LV- ratio: RV/LV ≥ 1.20.

**Table 7:** Sensitivity, specificity, positive predictive values and negative predictive values

Prediction models	Sensitivity (%)	Specificity (%)	PPV (%)	NPV (%)
*Model 1*				
PA/AA_AX_ ≥ 1	75	92	95	64
*Model 2*				
PA/AA_AX_ ≥ 1 or RV/LV_AX_ ≥ 1	100	48	80	100
PA/AA_AX_ ≥ 1 or RV/LV_AX_ ≥ 1.10	100	68	86	100
PA/AA_AX_ ≥ 1 or RV/LV_AX_ ≥ 1.15	98	76	89	95
PA/AA_AX_ ≥ 1 or RV/LV_AX_ ≥ 1.20	94	80	91	87
PA/AA_AX_ ≥ 1 or RV/LV_AX_ ≥ 1.30	94	84	92	88
*Model 3*				
PA/AA_AX_ ≥ 1 or RV/LV_4CH_ ≥ 1	100	40	77	100
PA/AA_AX_ ≥ 1 or RV/LV_4CH_ ≥ 1.10	100	68	86	100
PA/AA_AX_ ≥ 1 or RV/LV_4CH_ ≥ 1.15	98	76	89	95
PA/AA_AX_ ≥ 1 or RV/LV_4CH_ ≥ 1.20	96	80	91	91
PA/AA_AX_ ≥ 1 or RV/LV_4CH_ ≥ 1.30	94	84	92	88

*PPV* positive predictive value, *NPV* negative predictive value

## Discussion

In this study we tested different prediction models for precapillary PH using CTPA. Using an extensive statistical approach to obtain the best prediction model, we were able to show that combining ventricular and PA measurements (model 2 and 3) improved the predictive value of CTPA for precapillary PH.

Earlier studies mainly focussed on PA/AA_AX_ to predict PH and showed that a PA/AA_AX_ > 1 has a sensitivity and specificity ranging from 58–87 to 73–95 %, respectively [14, 15, 18–20]. This is in line with our results (PA/AA_AX_ > 1: sensitivity 75 % and specificity 92 %).

Multivariable binary logistic regression analyses and the significantly higher AUCs of model 2 and 3 compared to model 1, showed that there is a statistically significant improvement of the prediction model when ventricular and PA measurements are combined. DCA confirmed the clinical relevance of this approach. Arguing that, missing the diagnosis is worse than performing unnecessary diagnostic tests, we assigned a higher weight to false negatives than to false positives and focused on a range of low threshold probabilities. We showed that, even at this range of low threshold probabilities, in comparison to model 1, models 2 and 3 allowed a decrease in number of false positives without an increase in the number of false negatives. As such, adding ventricular measurements to PA measurements statistically improves the prediction model with clinical relevance.

We are aware of only one other study investigating ventricular measurements on CTPA to predict PH. Chan et al. [16] measured the RV/LV ratio in the axial view and found that a RV/LV > 1.28 predicted PH with a sensitivity of 85.7 and 86.1 %. There are no studies that used a combination of ventricular and pulmonary measurements to improve the predictive value of CTPA.

Manual reconstructed 4CH-views for determining ventricular diameters on standard CTPA have not been previously used in radiological studies of PH. In studies of patients of acute PE, some investigators indicated that the RV/LV determined in the 4 chamber view provided superior prediction of subsequent adverse events than the same ratio measured in the axial view, although other studies didn’t find any differences [18, 19, 23].

In this study, ROC analyses showed no significant difference between model 2 and 3 (*p* = 0.266) and also the net benefits determined with DCA were almost equal in both models. Therefore, determination of the RV/LV ratio in the axial view seems preferable as it does not require a manual reconstruction of the image.

We analyzed a range of cut-off values for the RV/LV ratio and did not use ROC analysis, as this method may not necessarily yield a clinically relevant cut-off value. To avoid missed diagnosis, the most suitable cut-off value for defining precapillary PH in this study was RV/LV ≥ 1.20 (model 2: sensitivity 94 %, specificity 80 %, PPV 91 %, NPV 87 %; model 3: sensitivity 96 %, specificity 80 %, PPV 91 %, NPV 91 %).

Recognizing the signs of PH on CTPA provides the radiologist with a tool to identify the disease timely. CTPA is often performed early in the diagnostic process of patients with unexplained dyspnea. Combining ventricular and PA measurements decreases the chance that the diagnosis of precapillary PH is missed. When there is suspicion of precapillary PH, and a CTPA is made, we recommend radiologists to assess not only the diameters of the great vessels, but also of both ventricles. When the PA/AA-ratio is greater or equal to 1 or when the RV/LV is greater or equal to 1.20, further diagnostic tests, to confirm or rule out PH are required. As a next diagnostic step, we would recommend to perform an echocardiography.

We want to emphasize, that CTPA measurements should not be used as a primary screening tool for precapillary PH. In isolation, CTPA measurements are not suitable to rule out or confirm the diagnosis of precapillary PH.

The reason for including patients with idiopathic pulmonary arterial hypertension and chronic tromboembolic PH in this analysis is that a timely diagnosis in these conditions can be lifesaving. Whether or not our results can be extrapolated to other forms of precapillary PH for which no treatment is currently available requires further investigations. In addition, we excluded patients with PH due to left sided systolic or diastolic heart failure (WHO group 2). That this may not be a major problem is suggested by the study of Chan et al. [16], in which mostly WHO group 2 PH patients were included and PA/AA_AX_ and RV/LV_AX_, measured separately, were good predictors of PH.

### Study limitations

First of all, baseline hemodynamic results suggested that all our PH patients were diagnosed in an advanced stage of their disease. We do not know whether our findings can be extrapolated to the earliest stages of the disease. Another limitation is that we performed a retrospective analysis. Preferably, a prospective analysis would be performed in a general population undergoing a CTPA for the evaluation of dyspnea. However, performing such a study would be very difficult regarding the low prevalence of precapillary PH.

18 CTPA studies were performed on a 4-slice CT system. Theoretically, on a 4-slice CT system, not all slices depicting the heart are in the same phase of the cardiac cycle. However, since the slices depicting the maximum diameter of the RV and LV were mostly adjacent or very close to each other, we did no experienced this problem.

## Conclusions

The predictive value of CTPA for precapillary PH improves when ventricular and PA measurements are combined. A PA/AA_AX_ ≥ 1 or a RV/LV_AX_ ≥ 1.20 needs further diagnostic evaluation to rule out or confirm the diagnosis.
